# Effectiveness and safety of modified fully oral 9-month treatment regimens for rifampicin-resistant tuberculosis: a prospective cohort study

**DOI:** 10.1016/S1473-3099(24)00228-7

**Published:** 2024-10

**Authors:** Oleksandr Korotych, Jay Achar, Elmira Gurbanova, Arax Hovhannesyan, Nino Lomtadze, Ana Ciobanu, Alena Skrahina, Gunta Dravniece, Liga Kuksa, Michael Rich, Naira Khachatryan, Myroslava Germanovych, Abdullat Kadyrov, Iana Terleieva, Irada Akhundova, Malik Adenov, Myahri Durdyeva, Nana Kiria, Nargiza Parpieva, Natalia Yatskevich, Rovshen Jumayev, Rustam Nurov, Saulius Diktanas, Valentina Vilc, Giovanni Battista Migliori, Askar Yedilbayev

**Affiliations:** a Division of Communicable Diseases, Environment, and Health, WHO Regional Office for Europe, Copenhagen, Denmark; b Department of Global Public Health, Karolinska Institutet, Stockholm, Sweden; c Department for Science and Innovation—National Research Fund Centre of Excellence for Biomedical Tuberculosis Research, South African Medical Research Council Centre for Tuberculosis Research, Division of Molecular Biology and Human Genetics, Faculty of Medicine and Health Sciences, Stellenbosch University, Cape Town, South Africa; d Lung Clinic, University of Tartu, Tartu, Estonia; e National Center for Tuberculosis and Lung Diseases, Tbilisi, Georgia; f The University of Georgia, Tbilisi, Georgia; g The Republican Scientific and Practical Center for Pulmonology and Tuberculosis, Minsk, Belarus; h PATH, Kyiv, Ukraine; i TB and Lung Disease Clinic, Riga East University Hospital, Riga, Latvia; j Partners In Health, Boston, MA, United States of America; k National Center of Pulmonology of the Ministry of Health of Armenia, Abovyan, Armenia; l State Institution “Public Health Center of the Ministry of Health of Ukraine”, Kyiv, Ukraine; m National Center of Phthisiology, Ministry of Health of Kyrgyzstan, Bishkek, Kyrgyzstan; n Scientific Research Institute of Lung Diseases, Baku, Azerbaijan; o National Scientific Center of Phthisiopulmonology of the Republic of Kazakhstan, Almaty, Kazakhstan; p Turkmen State Medical University, Ashgabat, Turkmenistan; q Republican Specialized Scientific-Practical Medical Center of Phthisiology And Pulmonology, Ministry of Health, Tashkent, Uzbekistan; r National Tuberculosis Treatment and Prevention Center, Directorate of Communicable Diseases, Ashgabat, Turkmenistan; s Republican Center for Protection of the Population from Tuberculosis, Ministry of Health and Social Protection of the Population, Dushanbe, Tajikistan; t Republican Klaipeda Hospital, Tuberculosis Branch, Klaipeda, Lithuania; u The Institute of Phthisiopneumology, Chisinau, Republic of Moldova; v Istituti Clinici Scientifici Maugeri, Istituto di Ricovero e Cura a Carattere Scientifico, Tradate, Italy

## Abstract

**Background:**

In 2020, WHO guidelines prioritised the use of a standard fully oral short treatment regimen (STR) consisting of bedaquiline, levofloxacin or moxifloxacin, ethionamide, ethambutol, high-dose isoniazid, pyrazinamide, and clofazimine for the management of rifampicin-resistant tuberculosis. A high prevalence of resistance to constituent drugs precluded its widespread use by countries in the WHO European region. We evaluated three 9-month fully oral modified STRs (mSTRs) in which ethionamide, ethambutol, isoniazid, and pyrazinamide were replaced by linezolid, cycloserine, or delamanid (or a combination).

**Methods:**

This multicountry, prospective, single-arm, cohort study examined the effectiveness and safety of mSTRs for fluoroquinolone-susceptible, rifampicin-resistant pulmonary tuberculosis in 13 countries in the WHO European region during 2020–23. We enrolled adults and children of all ages with bacteriologically confirmed rifampicin-resistant, fluoroquinolone-susceptible pulmonary tuberculosis, and children (aged 0–18 years) with clinically diagnosed disease and a confirmed contact with rifampicin-resistant, fluoroquinolone-susceptible tuberculosis. Participants aged 6 years or older received one of two regimens: bedaquiline, linezolid, levofloxacin, clofazimine, and cycloserine; or bedaquiline, linezolid, levofloxacin, clofazimine, and delamanid. Children younger than 6 years received delamanid, linezolid, levofloxacin, and clofazimine. Participants were followed up for 12 months after successful treatment completion to detect recurrence and death. The primary outcome was the cumulative probability of not having an unsuccessful study outcome (defined as treatment failure, on-treatment loss to follow-up, death, or recurrence) before 22 months of study follow-up. The primary safety outcome was the incidence of each adverse event of interest (peripheral neuropathy, optic neuritis, myelosuppression, hepatitis, prolonged QT interval, hypokalaemia, and acute kidney injury) of grade 3 or higher severity during the treatment course.

**Findings:**

Between Aug 28, 2020 and May 26, 2021, 7272 patients were screened and 2636 were included in the treatment cohort. 1966 (74·6%) were male, 670 (25·4%) were female, and median age was 43 years (IQR 33–53). Treatment success was recorded for 2181 (82·7%) participants. The cumulative probability of not having an unsuccessful study outcome 22 months after treatment initiation was 79% (95% CI 78–81). Increasing age (adjusted hazard ratio 2·61 [95% CI 1·70–4·04] for people aged >64 years *vs* 35–44 years), HIV-positive status (1·53 [1·16–2·01]), presence of bilateral cavities (1·68 [1·29–2·19]), smoking history (1·34 [1·05–1·71]), baseline anaemia (1·46 [1·15–1·86]), unemployment (1·37 [1·04–1·80]), elevated baseline liver enzymes (1·40 [1·13–1·73]), and excessive alcohol use (1·47 [1·14–1·89]) were positively associated with unsuccessful study outcomes. In the safety cohort of 2813 participants who received at least one dose, 301 adverse events of interest were recorded in 252 (9·0%) participants with the most frequent being myelosuppression (139 [4·9%] participants, 157 [52·2%] events).

**Interpretation:**

The high treatment success and good safety results indicate considerable potential for the use of mSTRs in programmatic conditions, especially for individuals not eligible for the current WHO-recommended 6-month regimen and in settings with a need for alternative options.

**Funding:**

The Global Fund to Fight AIDS, Tuberculosis and Malaria; United States Agency for International Development; Government of Germany; and WHO.

**Translation:**

For the Russian translation of the abstract see Supplementary Materials section.

**Research in context**

**Evidence before this study**

We searched PubMed for original research published between Jan 1, 2010 and Oct 31, 2023, that evaluated tuberculosis treatment regimens and reported end-of-treatment or end-of-follow-up outcomes. PubMed search terms used included “drug-resistant tuberculosis” AND “treatment” AND “short” OR “6-month” OR “9-month” AND “outcomes” AND “bedaquiline” OR “Bdq” AND “oral”, with no language restrictions. There was one trial reporting the efficacy of the combination of bedaquiline, linezolid, and levofloxacin of 6-month duration in comparison with a 9-month injection-based regimen among patients with multidrug-resistant (MDR) or rifampicin-resistant tuberculosis; one trial studying a 6-month regimen with the combination of bedaquiline, clofazimine, pyrazinamide, and levofloxacin prescribed for 28 weeks, supplemented by high-dose isoniazid and kanamycin for an 8-week intensive phase; one observational study investigating the effectiveness of a combination of bedaquiline, delamanid, linezolid, and clofazimine of 24–36 weeks duration among patients with pre-extensively drug-resistant (pre-XDR) tuberculosis; and one observational study of the effectiveness of a 9-month combination of bedaquiline, linezolid, levofloxacin, clofazimine, and cycloserine, but with a low sample size of 25 patients. Importantly, two trials (TB-PRACTECAL and ZeNix) reported on the effectiveness of regimens of 6-month duration consisting of bedaquiline, pretomanid, and linezolid with or without levofloxacin, which led to the 2023 changes in WHO policy guidance on the management of MDR or rifampicin-resistant tuberculosis and pre-XDR tuberculosis.

**Added value of this study**

This observational study provides the largest cohort study to date investigating the effectiveness and safety of 9-month all-oral treatment consisting of bedaquiline, linezolid, levofloxacin, clofazimine, and cycloserine or delamanid in patients with rifampicin-resistant tuberculosis with sensitivity to fluoroquinolones. With 83% of participants successfully treated and very few recurrences within 12-month follow-up, this 9-month modified short treatment regimen for rifampicin-resistant tuberculosis emerges as a practical alternative for individuals not eligible for the current WHO-recommended 6-month regimen, especially in settings with limited or no access to pretomanid.

**Implications of all the available evidence**

Shorter oral regimens characterised by favourable toxicity profiles hold promise for reducing rates of loss to follow-up. These regimens could also help to facilitate the WHO End TB Strategy goal of a 90% treatment success rate for all forms of tuberculosis by 2030.

## Introduction

In 2022, WHO estimated that 10·6 million people globally became ill with tuberculosis, representing a 4·5% increase from 2020.^1^ Tuberculosis is the world's second-leading infectious cause of death, the foremost cause of death in people living with HIV, and a major cause of mortality associated with antimicrobial resistance.^2^

The spread of drug-resistant tuberculosis, particularly multidrug-resistant (MDR) or rifampicin-resistant tuberculosis (defined as tuberculosis with resistance to both rifampicin and isoniazid, the two most potent antibiotics), constitutes a major public health threat, with 410 000 (3·9%) incident cases caused by MDR or rifampicin-resistant tuberculosis.^1^ Despite recent advances, MDR or rifampicin-resistant tuberculosis remains difficult to treat with reported success of 64%.^1^

In 2022, 16·3% of all global incident cases of MDR or rifampicin-resistant tuberculosis occurred in the WHO European region, despite this region accounting for only 2·2% of the global burden of tuberculosis.^1^ 85% of the MDR or rifampicin-resistant tuberculosis burden in this region originates within 18 countries in eastern Europe, central Asia, and the Baltics.^3^ In 2022, the prevalence of MDR or rifampicin resistance in the WHO European region was 24% among new cases and 54% among previously treated cases of tuberculosis, in contrast to the global prevalences of 3% and 17%, respectively.^1^ Treatment success in the region was reported in 55% of cases in 2020,^1^ significantly lower than the 80% target specified in 2025 regional milestones.^4^

Before 2019, injectable drugs had been the backbone of treatment regimens for MDR or rifampicin-resistant tuberculosis. WHO recommended their use for 4–8 months, with overall treatment duration up to 24 months.^5^ In 2020, WHO updated recommendations to deprioritise injectable drugs and include a new 9-month fully oral short treatment regimen (STR) consisting of bedaquiline, levofloxacin or moxifloxacin, ethionamide, ethambutol, high-dose isoniazid, pyrazinamide, and clofazimine for eligible patients with MDR or rifampicin-resistant tuberculosis without resistance to fluoroquinolones.^6^ In the WHO European region, the high prevalence of resistance to ethionamide, ethambutol, isoniazid, and pyrazinamide resulted in minimal programmatic adoption of the STR and continued use of longer alternatives.^7^

In 2019, to offer a locally tailored regimen and to generate data to support alternative fully oral STRs, the WHO Regional Office for Europe developed an operational study aiming to evaluate the effectiveness and safety of a modified STR (mSTR) in which ethionamide, ethambutol, isoniazid, and pyrazinamide were replaced by new and repurposed drugs, including linezolid, cycloserine, or delamanid (or a combination).

## Methods

### Study design

We did a prospective, single-arm, cohort study in 13 countries in the WHO European region. The study was approved by the WHO Research Ethics Review Committee and by individual countries' ethics committees (appendix 2 pp 6–7). The study is reported in line with STROBE guidelines.

### Participants

We enrolled participants older than 18 years with bacteriologically confirmed rifampicin-resistant pulmonary tuberculosis susceptible to fluoroquinolones, as assessed by culture-based or rapid molecular drug-susceptibility testing. Children (aged 0–18 years) with clinically diagnosed rifampicin-resistant pulmonary tuberculosis, based on history of close contact with an individual with confirmed rifampicin-resistant fluoroquinolone-susceptible tuberculosis, were also enrolled.

**Figure 1:**
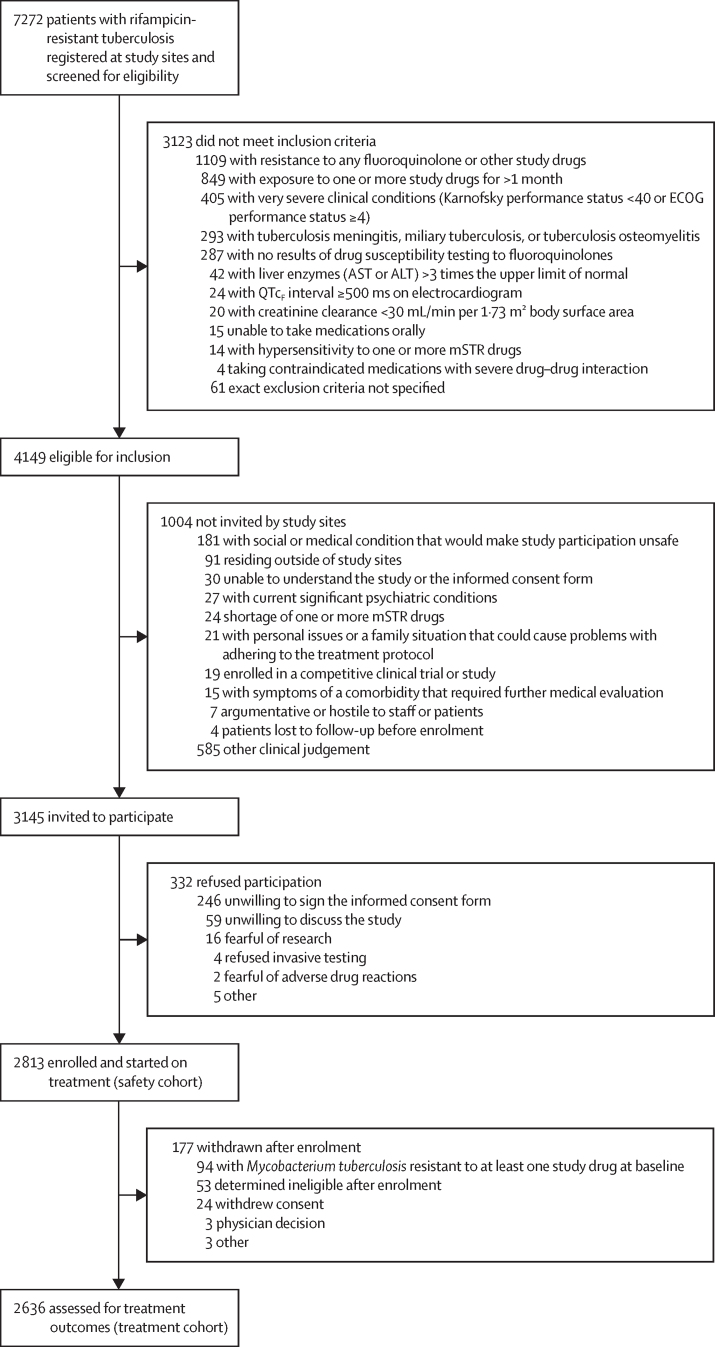
Study profile ALT=alanine aminotransferase. AST=aspartate aminotransferase. ECOG=Eastern Cooperative Oncology Group. mSTR=modified short treatment regimen. QTc_F_ interval=QT interval corrected with Fridericia's formula.

Exclusion criteria included documented resistance to mSTR components; previous treatment with mSTR drugs for at least 1 month; diagnosis of tuberculosis meningitis, miliary tuberculosis, or tuberculosis osteomyelitis; or presence of a very severe clinical condition (figure 1). Participants provided written informed consent. For children, consent was signed or witnessed by parents or legal guardians.

Geographical coverage of enrolment differed: five countries opted for nationwide roll-out, while others implemented study regimens in pilot regions. Historical data from patients treated with study regimens in Belarus and Georgia under research and routine programmatic conditions, respectively, between 2018 and 2021 were also included. All patients with bacteriologically confirmed rifampicin-resistant tuberculosis registered in participating regions during the study period (12 months per country) were screened for eligibility by district tuberculosis dispensaries. Patients starting treatment in hospitals and in outpatient settings were enrolled.

All participants who received at least one dose of a study treatment regimen were included in the safety cohort. The treatment cohort excluded withdrawn participants (those in whom exclusion criteria were encountered after inclusion and who did not satisfy criteria for unsuccessful study outcome). Withdrawn participants were referred for programmatic care.

### Procedures

Study regimens of 9-month duration were designed on the basis of knowledge of the drugs' safety and efficacy profiles detailed in WHO 2020 recommendations.^6^ In participants aged 6 years or older, two treatment regimens were available: bedaquiline, linezolid, levofloxacin, clofazimine, and cycloserine; or bedaquiline, linezolid, levofloxacin, clofazimine, and delamanid (in case of suspected resistance or intolerance to cycloserine). Children younger than 6 years received delamanid, linezolid, levofloxacin, and clofazimine.

Treatment was administered 7 days per week under direct observation or video support. Treatment normally consisted of 273 doses (allowed deviation 246–301 doses) within up to 43 weeks of treatment. Drug dosing was based on 2020 WHO recommendations (appendix 2 pp 8–9).

Clinical evaluations and blood and sputum mycobacteriology tests were done at baseline (0–30 days before treatment initiation), 2 weeks, and monthly during treatment (appendix 2 pp 11–12). Demographic characteristics and medical history were recorded on the basis of self-report and personal identification documents (for age and sex) at baseline. Participants who successfully completed treatment were followed up for 12 months to detect recurrence or death. At 3-month intervals, symptom screening and sputum mycobacteriology tests were done. Results of all evaluations were recorded in case record forms.

During treatment, participants were screened monthly for seven adverse events of interest of grade 3 or higher severity: peripheral neuropathy, optic neuritis, myelosuppression, hepatitis, prolonged QT interval (corrected with Fridericia's formula; QTc_F_), hypokalaemia, and acute kidney injury. Adverse events were graded using the endTB severity grading scale (version 5.0).

Data from case record forms and adverse event forms were entered into Epi Info version 7 (Centers for Disease Control and Prevention, Atlanta, GA, United States of America). Data quality was monitored quarterly, with online and face-to-face visits by the central study team and monitoring visits from country teams to study sites. The Virtual Medical Consilium established by the WHO Regional Office for Europe was responsible for reviewing and approving protocol deviations and advised study teams on the management of adverse events upon request.

### Outcomes

The primary effectiveness outcome was the cumulative probability of not having an unsuccessful study outcome before 22 months of follow-up (end-of-study outcome). An unsuccessful study outcome was defined as treatment failure, on-treatment loss to follow-up, death, or recurrence (appendix 2 p 13). Censoring of successfully treated participants occurred at the earlier of their last recorded follow-up appointment or 22 months after treatment initiation, to allow for participants who required additional time to complete treatment. The primary safety outcome was the incidence of each adverse event of interest (grade 3 or higher severity) during the treatment course.

Secondary outcomes were time to culture conversion among participants with positive baseline sputum Löwenstein–Jensen (LJ) culture (with conversion was defined as two consecutive valid negative LJ culture results more than 30 days apart); end-of-treatment outcome (appendix 2 p 18); and time to each adverse event of interest (grade ≥3) during treatment.

### Statistical analysis

Baseline participant characteristics and end-of-treatment outcomes are presented for the treatment cohort as counts and percentages or medians and IQRs. End-of-study outcomes are reported as cumulative probability with 95% CI. Treatment and study outcomes are also described by baseline HIV status. Crude effects of baseline CD4 count, HIV treatment regimen status, and co-trimoxazole prophylaxis status on study outcome and death are described in participants living with HIV.

The cumulative risk of culture conversion was calculated using the Kaplan–Meier method and was reported by 30-day increments until 120 days. Additionally, we report time to culture conversion using mycobacterial growth inhibitor tube culture exclusively or interchangeably with LJ culture (appendix 2 pp 2–3).

We described univariable associations between baseline characteristics and unfavourable study outcomes as crude hazard ratios (HRs), 95% CIs, and p values. We fitted a multivariable Cox proportional hazards model with a random intercept to account for clustering by country. Characteristics with p<0·1 on bivariable analysis with unsuccessful study outcome were included. Since very few values were missing, we used a complete-case approach for handling missing data. The effects of imputing missing values on model estimates were explored through sensitivity analyses.

For the safety analysis, we described the number and percentage of adverse events of interest of grade 3 or higher severity by terms, and median time to occurrence in the safety cohort. The incidence rates of adverse events of interest were computed as the total number of events divided by follow-up time, defined as the period from the date of treatment initiation to date of treatment outcome.

Statistical analyses were done with R software version 4.3.0.

### Role of the funding source

Among the funders of the study, only WHO had roles in study design, data collection, data analysis, data interpretation, and writing of the manuscript.

## Results

Participants were enrolled between Aug 28, 2020 and May 26, 2021. Of the 7272 patients screened at study sites, 4149 were eligible for inclusion, of whom 3145 were invited to participate and 1004 were not invited by decision of staff at individual study sites. The safety cohort included 2813 patients who agreed to participate, from 13 countries. After exclusion of 177 withdrawals, mainly due to baseline resistance (figure 1), 2636 participants were included in the treatment cohort. More than half of the participants were from Ukraine (1098 [41·7%]) or Belarus (540 [20·5%]).

Baseline demographic and clinical characteristics are described in table 1 and appendix 2 (pp 14–17). Median age was 43 years (IQR 33–53), with only 47 (1·8%) participants being younger than 15 years. Most participants were male (1966 [74·6%]). Of the 471 women aged 15–49 years, 441 (93·6%) had a baseline pregnancy test recorded, of which two (0·5%) were positive. A small proportion of participants reported injection drug use history (71 [2·7%]), homelessness (70 [2·7%]), and excessive alcohol use (415 [15·9%]). 1405 (53·5%) reported history of smoking. Evidence of HIV positivity (270 [10·2%]), hepatitis C virus infection (292 [11·7%]) and SARS-CoV-2 infection (83 [3·9%]) were present in a minority of participants. Baseline positive smear microscopy (1496 [58·2%]) and cavitary changes on x-ray (1518 [58·2%]) were detected in over half of participants, and most (1982 [75·2%]) had a positive baseline LJ culture result.

**Table 1:** Baseline demographic and clinical characteristics of participants in the treatment cohort

	**Participants (n=2636)**
**Age, years**	
Median (IQR)	43 (33–53)
<15	47/2636 (1·8%)
15–24	194/2636 (7·4%)
25–34	498/2636 (18·9%)
35–44	725/2636 (27·5%)
45–54	592/2636 (22·5%)
55–64	429/2636 (16·3%)
>64	151/2636 (5·7%)
**Sex**	
Male	1966/2636 (74·6%)
Female	670/2636 (25·4%)
**BMI, kg/m^2^**	
<18·5	580/2636 (22·0%)
≥18·5	2056/2636 (78·0%)
**Country**	
Armenia	28/2636 (1·1%)
Azerbaijan	101/2636 (3·8%)
Belarus	540/2636 (20·5%)
Georgia	100/2636 (3·8%)
Kazakhstan	148/2636 (5·6%)
Kyrgyzstan	93/2636 (3·5%)
Latvia	17/2636 (0·6%)
Lithuania	8/2636 (0·3%)
Republic of Moldova	104/2636 (3·9%)
Tajikistan	102/2636 (3·9%)
Turkmenistan	108/2636 (4·1%)
Ukraine	1098/2636 (41·7%)
Uzbekistan	189/2636 (7·2%)
**Inclusion cohort**	
Historical	333/2636 (12·6%)
Prospective	2303/2636 (87·4%)
**History of injection drug use**	
No	2550/2621 (97·3%)
Yes	71/2621 (2·7%)
Missing data	15
**Homeless**	
No	2555/2625 (97·3%)
Yes	70/2625 (2·7%)
Missing data	11
**Employment status**	
Employed	593/2636 (22·5%)
Unemployed	1603/2636 (60·8%)
Other	440/2636 (16·7%)
**History of incarceration**	
No	2373/2631 (90·2%)
Yes	258/2631 (9·8%)
Missing data	5
**Smoking history**	
No	1223/2628 (46·5%)
Yes	1405/2628 (53·5%)
Missing data	8
**Excessive alcohol use**	
No	2196/2611 (84·1%)
Yes	415/2611 (15·9%)
Missing data	25
**HIV-positive status**	
No	2363/2633 (89·7%)
Yes	270/2633 (10·3%)
Missing data	3
**HCV antibody status**	
Seronegative	2211/2503 (88·3%)
Seropositive	292/2503 (11·7%)
Missing data	133
**Diabetes**	
No	2415/2635 (91·7%)
Yes	220/2635 (8·3%)
Missing data	1
Baseline SARS-CoV-2 infection status*	
No	2036/2119 (96·1%)
Yes	83/2119 (3·9%)
Missing data	517
**Previous episode of tuberculosis**	
No	1983/2631 (75·4%)
Yes	648/2631 (24·6%)
Missing data	5
**X-ray cavities**	
No cavity	1113/2631 (42·3%)
Unilateral	1073/2631 (40·8%)
Bilateral	445/2631 (16·9%)
Missing data	5
**Baseline acid fast bacilli smear microscopy status**	
Negative	1085/2604 (41·7%)
Positive	1519/2604 (58·3%)
Missing data	32
**Treatment regimen**	
Levofloxacin, bedaquiline, linezolid, clofazimine, and cycloserine	2532/2636 (96·1%)
Levofloxacin, bedaquiline, linezolid, clofazimine, and delamanid	75/2636 (2·8%)
Levofloxacin, delamanid, linezolid, and clofazimine	29/2636 (1·1%)
**Baseline elevated AST or ALT**	
None	2065/2636 (78·3%)
Grade 1	516/2636 (19·6%)
Grade 2	45/2636 (1·7%)
Grade 3	9/2636 (0·3%)
Grade 4	1/2636 (<0·1%)
**Baseline peripheral neuropathy**	
None	2486/2636 (94·3%)
Grade 1	120/2636 (4·6%)
Grade 2	25/2636 (0·9%)
Grade 3	5/2636 (0·2%)
Grade 4	0/2636
**Baseline anaemia**	
None	2152/2636 (81·6%)
Grade 1	300/2636 (11·4%)
Grade 2	159/2636 (6·0%)
Grade 3	23/2636 (0·9%)
Grade 4	2/2636 (0·1%)
**Baseline renal dysfunction**	
None	2503/2636 (95·0%)
Grade 1	129/2636 (4·9%)
Grade 2	3/2636 (0·1%)
Grade 3	1/2636 (<0·1%)
Grade 4	0/2636
**Baseline visual loss**	
None	2022/2636 (76·7%)
Grade 1	181/2636 (6·9%)
Grade 2	237/2636 (9·0%)
Grade 3	133/2636 (5·0%)
Grade 4	63/2636 (2·4%)

Data are median (IQR) or n/N (%). ALT=alanine aminotransferase. AST=aspartate aminotransferase. HCV=hepatitis C virus.

* Based on PCR test.

At the end of treatment, 2181 (82·7%) participants were successfully treated and 113 (4·3%) died (appendix 2 p 18). Among 191 (7·2%) failed treatment episodes, reasons for failure were recorded for 133 (69·6%). The most frequently reported reasons were adverse events requiring treatment change (32 [24·1%]), clinician-driven treatment extensions (31 [23·3%]), and not receiving sufficient treatment (24 [18·0%]; appendix 2 p 19).

Of the 2181 participants who successfully completed treatment, 187 (8·6%) did not complete any post-treatment follow-up. The probability of not having a negative study outcome and remaining in follow-up was 87% (95% CI 85–88) after 6 months, 78% (77–80) after 11 months, and 33% (31–35) after 12 months of follow-up (appendix 2 p 4). During 12-month post-treatment follow-up, 22 (1·0%) recurrences and 44 (2·0%) additional deaths were recorded. The cumulative probability of not having an unsuccessful study outcome was 82% (95% CI 81–84) at 10 months, 80% (79–82) at 16 months, and 79% (78–81) at 22 months after treatment initiation (appendix 2 p 5).

Crude and adjusted associations between baseline characteristics and unsuccessful study outcomes are presented in table 2. In the adjusted analysis, increasing age, unemployment (adjusted HR 1·37 [95% CI 1·04–1·80]), excessive alcohol use (1·47 [1·14–1·89]), history of smoking (1·34 [1·05–1·71]), HIV-positive status (1·53 [1·16–2·01]), presence of bilateral pulmonary cavities on x-ray (1·68 [1·29–2·19]), elevated baseline aspartate aminotransferase or alanine aminotransferase (1·40 [1·13–1·73]), and baseline anaemia (1·46 [1·15–1·86]) were all positively associated with unsuccessful study outcomes. A BMI greater than 18·5 kg/m^2^ was negatively associated with an unsuccessful outcome (0·79 [0·63–0·97]). Positive associations between age and unsuccessful study outcomes increased in strength with increasing age: compared with participants aged 35–44 years, adjusted HRs were 1·21 (95% CI 0·94–1·56) in those aged 45–54 years, 1·38 (1·03–1·84) in those aged 55–64 years, and 2·61 (1·70–4·04) in those older than 64 years.

**Table 2:** Risk factors for unsuccessful study outcome from univariable and multivariable analysis in the treatment cohort

		**Crude**			**Adjusted**	
		Number of events	HR (95% CI)	p value*	HR (95% CI)†	p value*
Age, years		..	..	<0·0001	..	0·0003
	<15	2	0·19 (0·05–0·78)	0·021	0·32 (0·04–2·38)	0·26
	15–24	24	0·59 (0·38–0·91)	0·016	0·84 (0·49–1·45)	0·54
	25–34	84	0·83 (0·64–1·09)	0·18	1·07 (0·80–1·44)	0·65
	35–44	145	1 (ref)	..	1 (ref)	..
	45–54	130	1·10 (0·87–1·39)	0·44	1·21 (0·94–1·56)	0·14
	55–64	93	1·09 (0·84–1·41)	0·52	1·38 (1·03–1·84)	0·031
	>64	43	1·51 (1·08–2·12)	0·017	2·61 (1·70–4·04)	<0·0001
Sex		..	..	0·020	..	0·86
	Male	409	1 (ref)	..	1 (ref)	..
	Female	112	0·79 (0·64–0·97)	0·023	1·03 (0·80–1·32)	0·81
BMI, kg/m^2^		..	..	0·0003	..	0·026
	<18·5	144	1 (ref)	..	1 (ref)	..
	≥18·5	377	0·69 (0·57–0·84)	0·0002	0·79 (0·63–0·97)	0·026
History of injection drug use		..	..	0·053	..	0·35
	No	492	1 (ref)	..	1 (ref)	..
	Yes	20	1·61 (1·03–2·51)	0·038	0·80 (0·47–1·37)	0·41
Homeless		..	..	0·029	..	0·65
	No	500	1 (ref)	..	1 (ref)	..
	Yes	21	1·69 (1·09–2·62)	0·018	0·91 (0·57–1·48)	0·71
Employment status		..	..	<0·0001	..	0·082
	Employed	80	1 (ref)	..	1 (ref)	..
	Unemployed	364	1·81 (1·42–2·30)	<0·0001	1·37 (1·04–1·80)	0·023
	Other	77	1·34 (0·98–1·84)	0·065	1·14 (0·77–1·67)	0·51
History of incarceration		..	..	0·050	..	0·35
	No	456	1 (ref)	..	1 (ref)	..
	Yes	63	1·32 (1·01–1·71)	0·043	1·13 (0·83–1·53)	0·44
Smoking history		..	..	<0·0001	..	0·044
	No	173	1 (ref)	..	1 (ref)	..
	Yes	346	1·89 (1·58–2·27)	<0·0001	1·34 (1·05–1·71)	0·020
Excessive alcohol use		..	..	<0·0001	..	0·0016
	No	393	1 (ref)	..	1 (ref)	..
	Yes	118	1·69 (1·37–2·07)	<0·0001	1·47 (1·14–1·89)	0·0028
HIV-positive status		..	..	<0·0001	..	0·0051
	No	426	1 (ref)	..	1 (ref)	..
	Yes	94	2·21 (1·77–2·77)	<0·0001	1·53 (1·16–2·01)	0·0023
HCV antibody status			..	<0·0001	..	0·53
	Seronegative	407	1 (ref)	..	1 (ref)	..
	Seropositive	83	1·67 (1·32–2·11)	<0·0001	1·10 (0·84–1·45)	0·48
Diabetes		..	..	0·67	..	..
	No	473	1 (ref)	..	..	..
	Yes	47	1·07 (0·79–1·44)	0·67	..	..
Baseline SARS-CoV-2 infection status‡		..	..	0·71	..	..
	No	410	1 (ref)	..	..	..
	Yes	18	1·09 (0·68–1·76)	0·71	..	..
Previous tuberculosis episode		..	..	0·059	..	0·58
	No	375	1 (ref)	..	1 (ref)	..
	Yes	145	1·21 (1·00–1·46)	0·056	1·06 (0·86–1·30)	0·60
X-ray cavities		..	..	<0·0001	..	0·0005
	No cavity	167	1 (ref)	..	1 (ref)	..
	Unilateral	221	1·43 (1·17–1·74)	0·0005	1·24 (0·98–1·56)	0·072
	Bilateral	132	2·20 (1·75–2·77)	<0·0001	1·68 (1·29–2·19)	0·0001
Baseline smear microscopy status		..	..	<0·0001	..	0·052
	Negative	169	1 (ref)	..	1 (ref)	..
	Positive	346	1·54 (1·28–1·85)	<0·0001	1·22 (0·98–1·52)	0·077
Treatment regimen		..	..	0·099	..	..
	Levofloxacin, bedaquiline, linezolid, clofazimine, and cycloserine	501	1 (ref)	..	..	..
	Levofloxacin, bedaquiline, linezolid, clofazimine, and delamanid	18	1·24 (0·77–1·98)	0·38	..	..
	Levofloxacin, delamanid, linezolid, and Clofazimine	2	0·32 (0·08–1·30)	0·11	..	..
Baseline elevated AST or ALT		..	..	<0·0001	..	0·0021
	No	368	1 (ref)	..	1 (ref)	..
	Yes	153	1·63 (1·35–1·96)	<0·0001	1·40 (1·13–1·73)	0·0019
Baseline peripheral neuropathy		..	..	0·063	..	0·94
	No	482	1 (ref)	..	1 (ref)	..
	Yes	39	1·38 (1·00–1·91)	0·052	0·98 (0·68–1·42)	0·93
Baseline anaemia		..	..	0·0019	..	0·0029
	No	400	1 (ref)	..	1 (ref)	..
	Yes	121	1·40 (1·14–1·71)	0·0013	1·46 (1·15–1·86)	0·0019
Baseline renal dysfunction		..	..	0·056	..	0·51
	No	487	1 (ref)	..	1 (ref)	..
	Yes	34	1·43 (1·01–2·02)	0·044	1·16 (0·79–1·69)	0·45
Baseline visual loss		..	..	0·38	..	..
	No	405	1 (ref)	..	..	..
	Yes	116	0·91 (0·74–1·12)	0·38	..	..

N values indicate the total number of participants with available data and included in the analyses. ALT=alanine aminotransferase. AST=aspartate aminotransferase. HCV=hepatitis C virus. HR=hazard ratio.

* The Wald test was used to calculate p values for categories within each independent variable, while the likelihood ratio test, based on the log partial likelihood, was used to calculate p values for each independent variable overall.

† Variables were selected for inclusion in the adjusted model where the p value related to the crude association was <0·1.

‡ Based on PCR test.

Baseline CD4 count was available for 245 (90·7%) of 270 participants living with HIV. In this group, median baseline CD4 count was 172 cells per mm^3^ (IQR 71–350). 233 (86·3%) participants living with HIV were receiving antiretroviral therapy (ART) at baseline, among whom dolutegravir-based regimens were most frequently used (211 [90·6%]). 254 (97·0%) of 262 participants living with HIV were receiving co-trimoxazole.

End-of-treatment responses were less favourable among participants living with HIV: 187 (69·5%) of 269 had successful treatment compared with 1992 (84·3%) of 2363 people without HIV. Death (29 [10·8%] of 269 with available data *vs* 84 [3·6%] of 2363) and loss to follow-up (29 [10·8%] of 269 *vs* 121 [5·1%] of 2363) were also more frequent in participants living with HIV than in those without HIV (appendix 2 p 20).

Among participants living with HIV, there was moderate evidence of a positive crude association between not receiving co-trimoxazole and unsuccessful study outcome (HR 2·69 [95% CI 1·09–6·63]) or death (3·80 [1·17–12·40]; appendix 2 p 21). Compared with having a CD4 count of 100 cells per mm^3^ or lower, having a CD4 count higher than 500 cells per mm^3^ showed a moderate negative association with unsuccessful study outcome (0·40 [0·17–0·96]) or death (0·23 [0·05–0·99]). Receiving ART showed a crude negative association with death (0·43 [0·20–0·90]).

The cumulative probability of culture conversion using LJ culture was 28% (IQR 26–30) after 30 days, 55% (53–57) after 60 days, 68% (66–70) after 90 days, and 73% (70–75) after 120 days of treatment. Median time to sputum culture conversion was 57 days (IQR 55–59; appendix 2 p 1).

Among 2813 participants in the safety cohort, 301 adverse events of interest (grade ≥3 severity) were reported, affecting 252 (9·0%) participants and resulting in a cumulative incidence rate of 1·32 (95% CI 1·18–1·48) adverse events of interest per 100 person-months (table 3). 212 (7·5%) participants had only one adverse event of interest, 32 (1·1%) had two, and eight (0·2%) had three or more.

**Table 3:** Summary of adverse events of interest of grade 3 or higher during treatment among patients in the safety cohort

	Participants with adverse events of interest*(n=2813)	**Adverse events of interest**†**(n=301)**	**Median days to adverse event of interest**	**Rate per 100 person-months**
Total	252 (9·0%)	301	98 (56–154)	1·32 (1·18–1·48)
Myelosuppression	139 (4·9%)	157 (52·2%)	90 (54–132)	0·69 (0·59–0·80)
QTc_F_ interval prolongation	48 (1·7%)	49 (16·3%)	104 (51–154)	0·21 (0·16–0·28)
Peripheral neuropathy	31 (1·1%)	32 (10·6%)	137·5 (96·5–177)	0·14 (0·10–0·20)
Hepatitis	24 (0·9%)	25 (8·3%)	101 (46–182)	0·11 (0·74–0·16)
Optic neuritis	16 (0·6%)	16 (5·3%)	144 (98·5–179)	0·07 (0·04–0·11)
Acute kidney injury	13 (0·5%)	15 (5·0%)	37 (29–120)	0·07 (0·04–0·11)
Hypokalaemia	7 (0·2%)	7 (2·3%)	136 (63–229)	0·03 (0·01–0·06)

Data are n (%), median (IQR), or rate (95% CI). QTc_F_ interval =QT interval corrected with Fridericia's formula.

* Includes participants with at least one episode of each adverse event of interest of grade 3 or higher; percentages are calculated from the total number of patients in the safety cohort.

† Percentages are calculated from the total number of adverse events of interest.

The three most frequent adverse events of interest (grade 3 or higher) were myelosuppression (affecting 139 [4·9%] people with 157 events), QTc_F_ interval prolongation (48 [1·7%] people with 49 events), and peripheral neuropathy (31 [1·1%] people with 43 events; table 3). Myelosuppression accounted for 52·2% of all adverse events of interest. The median time to adverse event of interest was 98 days (IQR 56–154). By onset of occurrence, the earliest events were acute kidney injury, myelosuppression, and hepatitis, and the latest were peripheral neuropathy, hypokalaemia, and optic neuritis (table 3).

**Figure 2:**
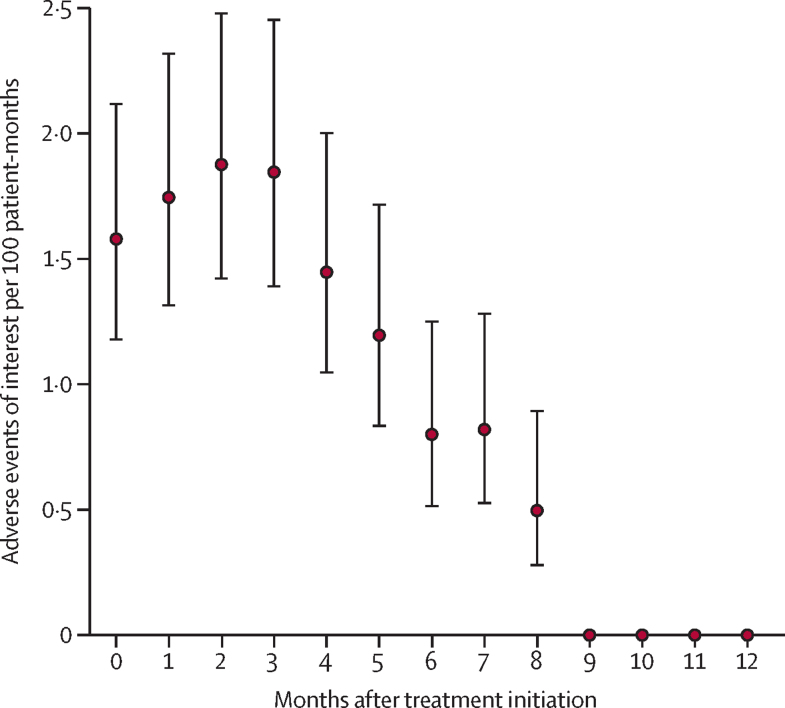
Incidence rate of adverse events of interest per month after treatment initiation Error bars are 95% CIs. Adverse events of interest were peripheral neuropathy, optic neuritis, myelosuppression, hepatitis, prolonged QT interval (corrected with Fridericia's formula), hypokalaemia, and acute kidney injury, all of grade 3 or higher.

The risk of grade three or higher adverse events of interest peaked at the third month of treatment, reaching 1·9 (95% CI 1·4–2·5) events per 100 person-months with linear gradual decline until end of treatment (figure 2).

## Discussion

We report the effectiveness and safety of fully oral mSTRs for patients with MDR or rifampicin-resistant, fluoroquinolone-susceptible tuberculosis, within the largest prospective study in the WHO European region, where the burden of MDR or rifampicin-resistant tuberculosis is high. The study was conducted before 2023 WHO recommendation updates that shortened treatment for MDR or rifampicin-resistant tuberculosis to 6 months.

We found that end-of-treatment success rates with mSTRs were higher than those of the WHO-recommended^8^ fully oral STR (74%), reported in an observational cohort study in South Africa,^9^ and the 9-month all-oral regimen containing delamanid, linezolid, levofloxacin, and pyrazinamide (78%), studied in a randomised controlled trial in South Korea.^10^ The cumulative probability of not having an unsuccessful study outcome 22 months after treatment initiation with an mSTR was also higher than the sustained treatment success with either the WHO-recommended STR or delamanid-based 9-month regimen. The median time to culture conversion was longer than that under the 9-month delamanid-based regimen (27 days), but the consistency of recording of culture results in our study was suboptimal due to programmatic challenges. Importantly, mortality risk in our study was lower than that reported with the WHO-recommended STR (24%) and similar to that of the delamanid-based 9-month regimen (6%), potentially due to better tolerability. Unlike our study, the TB-PRACTECAL^11^ study, evaluating regimens containing bedaquiline, pretomanid, linezolid, and moxifloxacin (BPaLM), did not report any deaths, potentially because of the small cohort size (151 participants) and highly selective nature of study inclusion criteria. The recurrence rate in our study was similar to that with BPaLM (0·0%), the WHO-recommended STR (0·2%), and the 9-month delamanid-based regimen (1·6%).^10^, ^11^

Although the treatment success rate in our study, conducted under close-to-programmatic conditions, is lower than that reported in a clinical trial involving BPaLM treatment (89%), it slightly surpasses the real-world performance of BPaLM (82%).^11^, ^12^ Furthermore, since the medicines included in our study are recommended for use without age restrictions and during pregnancy or breastfeeding, mSTRs could be considered complementary to BPaLM or could be used in place of BPaLM in settings with no access to pretomanid.^11^, ^13^

In our study, HIV-positive status was associated with significantly lower treatment success, which was not found in data from South Africa or TB-PRACTECAL.^9^, ^11^ One explanation is that our study did not exclude people with HIV who were not receiving ART or co-trimoxazole preventive therapy or those on ART with low CD4 counts at baseline. Overall, the WHO European region is characterised by late diagnosis of HIV and HIV-associated tuberculosis, and poor outcomes in more than half of tuberculosis cases in people living with HIV.^14^, ^15^

We found that smoking and excessive alcohol use adversely affected treatment success. Previous research has indicated that excessive alcohol consumption is linked to increased rates of relapse and death among patients with tuberculosis.^16^ This association might be attributable to the interaction of alcohol with the metabolism of antituberculosis drugs, elevating the risk of hepatotoxic and cardiotoxic reactions, reduced treatment adherence, and impaired pulmonary innate immune function.^17^, ^18^ Smoking has been linked to delayed culture conversion and treatment response.^19^, ^20^

Older adults (particularly those aged 55–64 years or >64 years) were at higher risk for poor treatment outcomes. This risk might be due to immunosenescence and comorbidities, highlighting the importance of age-appropriate tuberculosis care.^21^ Study findings also outlined the importance of addressing undernutrition as a factor significantly associated with poor outcomes.^22^ Nutritional support substantially reduces the risks of tuberculosis mortality.^23^ Moderate or severe anaemia at baseline was identified as another risk factor for poor outcomes. While anaemia in patients with tuberculosis typically results from nutritional deficiencies and the anaemia of chronic disease, it can be exacerbated by the haematological toxicity associated with linezolid.^24^

The presence of bilateral cavities in the lungs decreased the chance of successful treatment. Although the clinical trial of BPaLM did not show variation in treatment success related to the presence of cavities, real-world BPaLM outcomes indicate diminished sputum culture conversion in such patients.^12^ This observation might be explained by decreased drug penetration into cavity lesions.^25^ The best way to prevent the development of extensive disease is early case identification and rapid initiation of effective treatment.

The findings of our study on safety were consistent with other studies evaluating shorter fully oral regimens.^11^, ^26^, ^27^, ^28^ Similar to our findings, severe myelosuppression occurred among 3% (TB-PRACTECAL trial),^11^ 4·4% (ZeNix trial),^26^ and 7·4% (endTB trial)^27^ of participants in other studies. Severe QTc_F_ prolongation affected 1% of participants in the TB-PRACTECAL trial (the second most frequent grade 3 or higher adverse event in this study), 2% in the ZeNix trial, 1·5% in the endTB trial, and 3% in the STREAM stage 2 trial.^28^ None of the peripheral neuropathy events observed in the TB-PRACTECAL or ZeNix studies were of severity grade 3–4, while the endTB trial reported 3·4% incidence among the five endTB intervention groups, which is higher than that observed within the present cohort (1·1%).^11^, ^26^, ^27^

The peak of severe adverse events of interest in the third month of treatment provides a crucial temporal insight that might inform clinical monitoring practices and patient-management strategies. Median times to grade 3 or higher adverse events ranged across adverse events from 1·9 to 5·8 months within the endTB trial.^27^ The TB-PRACTECAL and ZeNix trials did not report on the time to adverse events. Our findings contribute to a growing body of evidence that reinforces the therapeutic potential of these regimens and underscores the need for vigilant monitoring of adverse events of interest to mitigate risks and enhance patient safety.

The trajectory of treatment regimens for drug-resistant tuberculosis appears promising, with ongoing efforts directed towards the inclusion of populations at risk (children and people who are pregnant or breastfeeding), improving regimen tolerability and participant retention, and preventing drug resistance.^29^ We believe that our study contributes to advancing these crucial aspects of rifampicin-resistant tuberculosis management.

The large cohort size, multinational design, few exclusion criteria (particularly with respect to age and HIV status), and treatment provision at close-to-programmatic conditions are all important strengths of our study. However, the lack of a randomised control group is an important limitation that precludes comparisons with programmatically available regimens. Additional limitations include non-inclusion of a portion of patients by study site based on clinical judgement (figure 1) and post-treatment loss to follow-up. A large proportion of those lost to follow-up after treatment success were treated in Ukraine, a country heavily affected by the full-scale war against it during study implementation. However, we did a sensitivity analysis excluding Ukrainian data and found that post-treatment loss to follow-up had little influence on end-of-study success estimates (data not shown).

Future research should focus on designing and testing psychosocial interventions, such as cessation of smoking and alcohol consumption, to support patients at risk of unsuccessful outcomes. Patient and clinician acceptability of various shorter treatment options should also be explored in qualitative or mixed-methods investigations. Further analysis of our cohort will focus on describing incidence, time to occurrence, actions taken, outcomes, and risk factors for serious adverse events and adverse events of interest of grade 3 or higher.

High rates of treatment success and high probability of not having unsuccessful study outcomes indicate good potential for use of an mSTR under programmatic conditions. The three regimens evaluated here will broaden national tuberculosis programmes' regimen choices, particularly for individuals not eligible for the current WHO-recommended 6-month regimen (ie, people who are pregnant or breastfeeding, and children aged <14 years) and in settings with limited access to pretomanid. These mSTRs could help to facilitate WHO's End TB Strategy goal of a 90% treatment success rate for all forms of tuberculosis by 2030.

For the **endTB severity grading scale** see https://endtb.org/resources/pharmacovigilance

### Contributors

### Data sharing

De-identified participant data and a data dictionary will be made available through the TB-IPD platform from July, 2024, upon reasonable request, which will be reviewed by a data access committee. Study related documents (including the protocol, case record forms, and patient consent forms) are available upon request from the corresponding author.

## Declaration of interests

We declare no competing interests.

## Supplementary Materials

Russian translation of the abstract
