# Supplementary material for: Effectiveness and safety of modified fully oral 9-month treatment regimens for rifampicin-resistant tuberculosis: a prospective cohort study
Source: Lancet Infect Dis. 2024 Oct;24(10):1151–61. doi: 10.1016/S1473-3099(24)00228-7 (PMC11424498; doi:10.1016/S1473-3099(24)00228-7)
Supplement: Russian translation of the abstract [file mmc1.pdf]

THE LANCET
Infectious Diseases

Supplementary appendix 1

This translation in Russian was submitted by the authors and we reproduce it as supplied. It has not been peer reviewed. *The Lancet*'s editorial processes have only been applied to the original in English, which should serve as reference for this manuscript.

Этот перевод на русский язык был предоставлен авторами, и мы воспроизводим его в том виде, в котором он был предоставлен. Этот перевод не был рецензирован. Редакционные процессы журнала The Lancet были применены только к оригиналу на английском языке, который служит отсылкой для этой статьи.

Supplement to: Korotych O, Achar J, Gurbanova E, et al. Effectiveness and safety of modified fully oral 9-month treatment regimens for rifampicin-resistant tuberculosis: a prospective cohort study. *Lancet Infect Dis* 2024; published online June 13. https://doi.org/10.1016/S1473-3099(24)00228-7.

Аннотация

Предыстория

В 2020 г. руководство ВОЗ определило приоритетность использования стандартного безынъекционного краткосрочного режима лечения (КРЛ), состоящего из бедаквилина, левофлоксацина или моксифлоксацина, этионамида, высоких доз изониазида, пиразинамида и клофазимина, для туберкулеза с лекарственной устойчивостью возбудителя к рифампицину. Высокая распространенность лекарственной устойчивости к входящим в состав режима противотуберкулезным препаратам препятствовала его широкому применению в странах Европейского региона ВОЗ. Мы провели оценку трех 9-месячных модифицированных безынъекционных КРЛ (мКРЛ), в которых этионамид, этамбутол, изониазид и пиразинамид были заменены на линезолид, циклосерин или деламанид (или их комбинацию).

Методы

В данном многострановом проспективном несравнительном когортном исследовании изучались эффективность и безопасность мКРЛ для лечения туберкулеза легких с сохраненной чувствительностью возбудителя к фторхинолонам и лекарственной устойчивостью к рифампицину в 13 странах Европейского региона ВОЗ в период 2020–2023 гг. В исследование были включены взрослые и дети всех возрастов с бактериологически подтвержденным туберкулезом легких с лекарственной устойчивостью к рифампицину и сохраненной чувствительностью к фторхинолонам, а также дети (в возрасте 0–18 лет) с клинически диагностированным заболеванием и подтвержденным контактом с больным туберкулезом с лекарственной устойчивостью к рифампицину и сохраненной чувствительностью к фторхинолонам. Участники в возрасте от 6 лет и старше получали один из двух режимов: бедаквилин, линезолид, левофлоксацин, клофазимин и циклосерин или бедаквилин, линезолид, левофлоксацин, клофазимин и деламанид. Дети младше 6 лет получали деламанид, линезолид, левофлоксацин и клофазимин. Последующее наблюдение за участниками осуществлялось на протяжении 12 месяцев после успешного завершения лечения для выявления случаев повторного заболевания и смерти. Первичным результатом была совокупная вероятность отсутствия неблагоприятного исхода исследования (определяемого как неудача лечения, потеря для последующего наблюдения в ходе лечения, смерть или повторное заболевание) на протяжении 22 месяцев наблюдения. Первичным результатом оценки безопасности считалась частота возникновения во время лечения каждого из представляющих интерес нежелательных явлений (периферическая нейропатия, неврит зрительного нерва, миелосупрессия, гепатит, удлинение интервала QT, гипокалиемия и острое повреждение почек) 3-й степени тяжести или выше.

Результаты

С 28 августа 2020 г. по 26 мая 2021 г. 7272 пациента прошли скрининг, и 2636 из них были включены в когорту лечения. 1966 человек (47,6%) были мужчинами, 670 (25,4%) – женщинами, медиана возраста составила 43 года (МКР 33–53). Успех лечения был зарегистрирован у 2181 (82,7%) участника. Совокупная вероятность отсутствия неблагоприятного исхода исследования через 22 месяца после начала лечения составила 79% (95% ДИ 78–81). Увеличение возраста (скорректированное отношение рисков 2,61 [95% ДИ 1,70–4,04] для людей старше 64 лет по сравнению с группой в возрасте 35– 44 лет), ВИЧ-положительный статус (1,53 [1,16–2,01]), наличие двусторонних полостей распада (1,68 [1,29–2,19]),

курение в анамнезе (1,34 [1,05–1,71]), наличие анемии до начала лечения (1,46 [1,15–1,86]), статус безработного (1,37 [1,04–1,80]), повышение уровня ферментов печени до начала лечения (1,40 [1,13–1,73]) и чрезмерное употребление алкоголя (1,47 [1,14–1,89]) были положительно связаны с неблагоприятными исходами исследования. В когорте оценки безопасности режимов, состоящей из 2813 участников, получивших как минимум одну дозу препаратов, у 252 (9,0%) человек было зафиксировано 301 нежелательное явление, представляющее интерес, наиболее частым из которых была миелосупрессия (139 [4,9%] участников, 157 [52,2%] явлений). Интерпретация Высокие результаты успешности лечения и хорошие показатели безопасности свидетельствуют о значительном потенциале использования мКРЛ в программных условиях, особенно для лечения людей, не соответствующих критериям назначения рекомендованного в настоящее время ВОЗ 6-месячного режима, а также в условиях, когда требуются альтернативные варианты.

Финансирование

Глобальный фонд для борьбы со СПИДом, туберкулезом и малярией; Агентство США по международному развитию; Правительство Германии; ВОЗ.

Авторское право

© 2024 г. Всемирная организация здравоохранения. Опубликовано компанией Elsevier Ltd. Все права защищены. Данная статья находится в открытом доступе и опубликована на условиях лицензии CC BY 3.0 IGO, разрешающей неограниченное использование, распространение и воспроизведение на любом носителе при условии надлежащего цитирования оригинальной работы. При любом использовании данной статьи не должно создаваться впечатление, что ВОЗ одобряет какую-либо конкретную организацию, продукты или услуги. Использование логотипа ВОЗ не допускается. Данное уведомление должно быть сохранено вместе с оригинальным URL-адресом статьи.
