# Supplementary material for: Effectiveness and safety of modified fully oral 9-month treatment regimens for rifampicin-resistant tuberculosis: a prospective cohort study
Source: Lancet Infect Dis. 2024 Oct;24(10):1151–61. doi: 10.1016/S1473-3099(24)00228-7 (PMC11424498; doi:10.1016/S1473-3099(24)00228-7)
Supplement: Supplementary appendix 2 [file mmc2.pdf]

THE LANCET
Infectious Diseases

Supplementary appendix 2

This appendix formed part of the original submission and has been peer reviewed. We post it as supplied by the authors.

Supplement to: Korotych O, Achar J, Gurbanova E, et al. Effectiveness and safety of modified fully oral 9-month treatment regimens for rifampicin-resistant tuberculosis: a prospective cohort study. *Lancet Infect Dis* 2024; published online June 13. https://doi.org/10.1016/S1473-3099(24)00228-7.

Figure 1. Kaplan Meier estimates for time to sputum culture conversion using Löwenstein-Jensen culture.

Characteristic	Median, days	30 days	60 days	90 days	120 days
Overall	57 (55, 59)	28% (26%, 30%)	55% (53%, 57%)	68% (66%, 70%)	73% (70%, 75%)

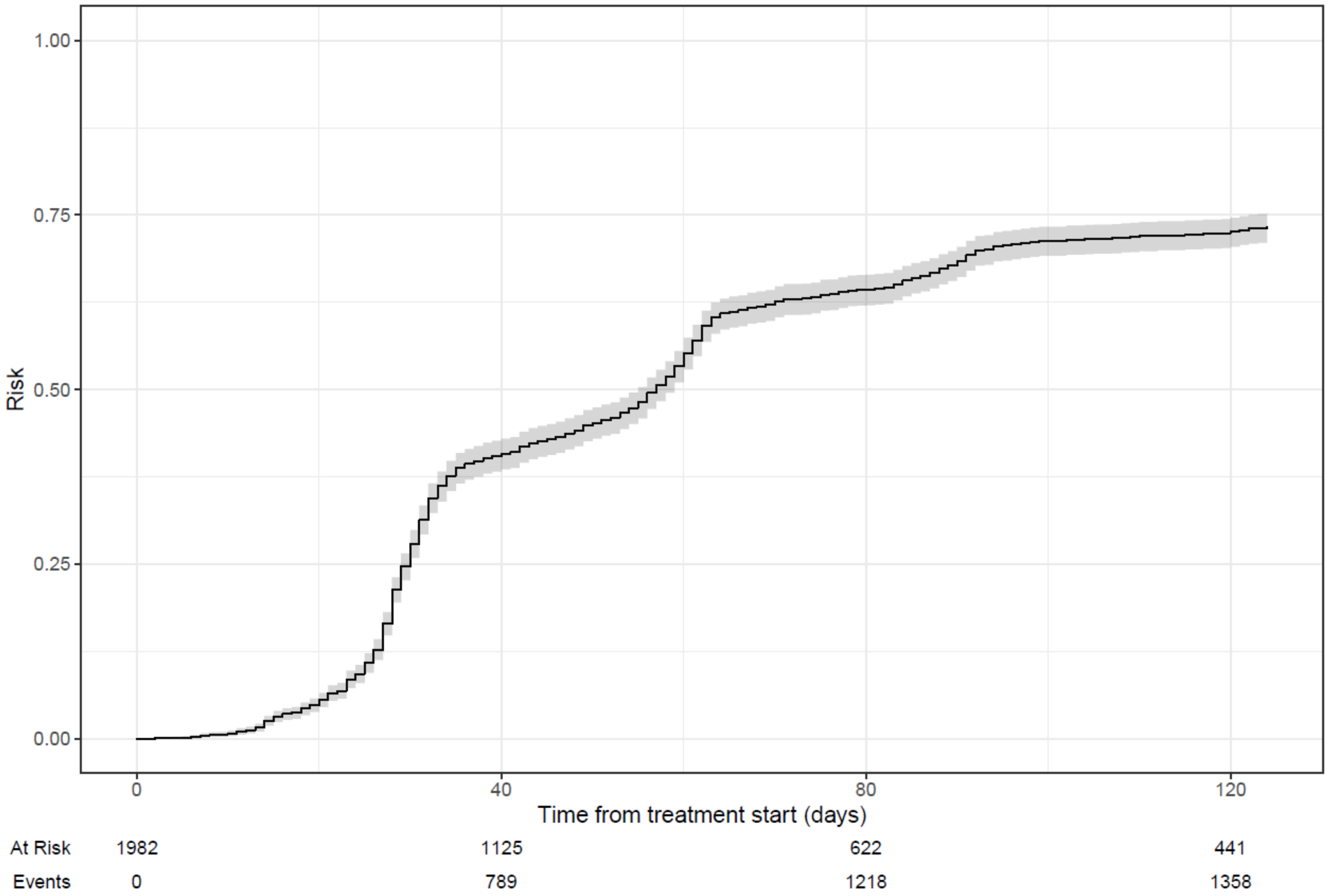

Figure 2. Kaplan Meier estimates for time to sputum culture conversion using Mycobacterial Growth Inhibitor Tube culture.

Characteristic	Median, days	30 days	60 days	90 days	120 days
Overall	86 (71, 90)	19% (18%, 21%)	41% (38%, 43%)	53% (50%, 55%)	58% (55%, 60%)

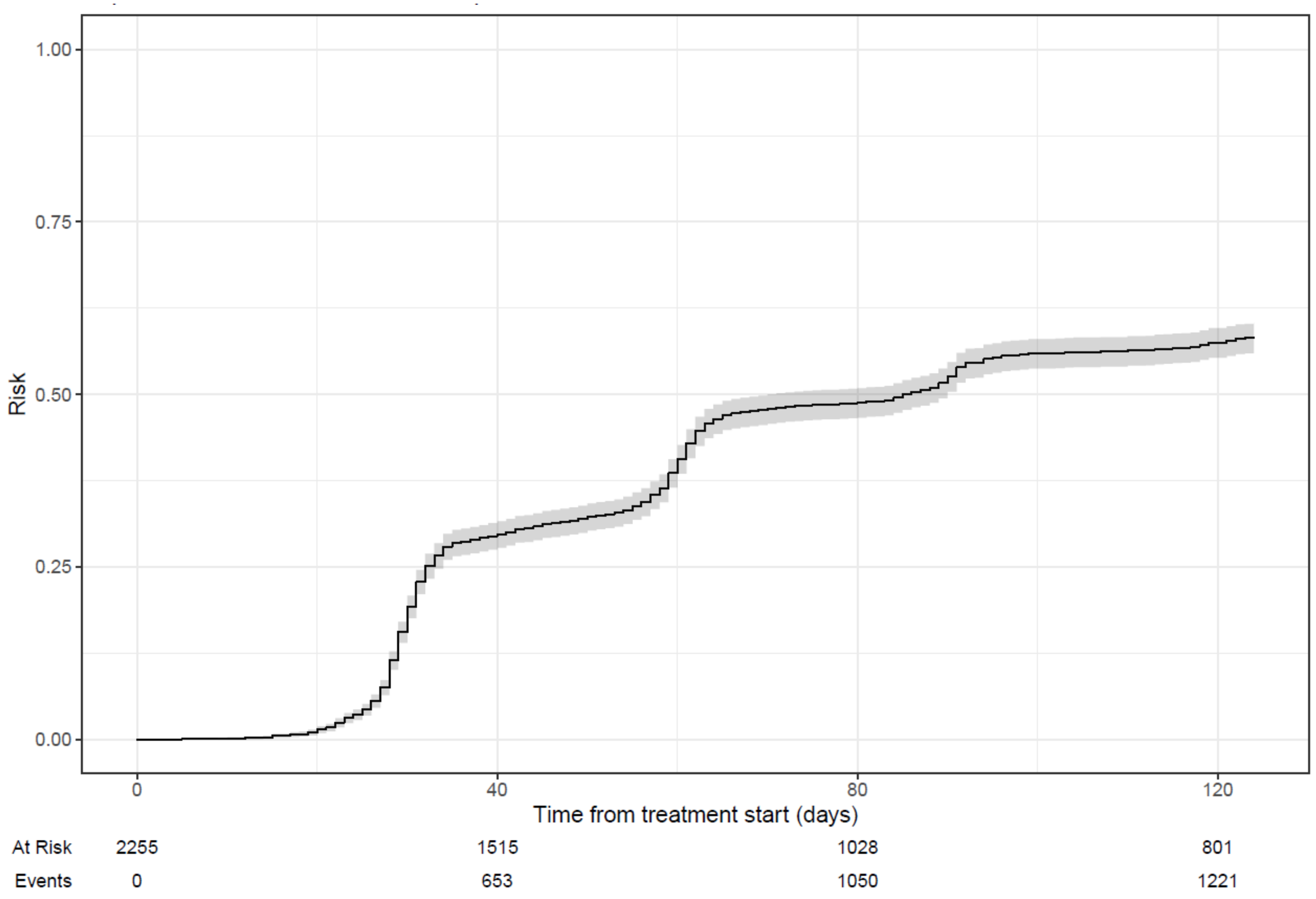

Figure 3. Kaplan Meier estimates for time to sputum culture conversion using Mycobacterial Growth Inhibitor Tube and Löwenstein-Jensen cultures interchangeably.

Characteristic	Median, days	30 days	60 days	90 days	120 days
Overall	34 (33, 35)	37% (35%, 39%)	72% (70%, 74%)	89% (88%, 90%)	95% (93%, 96%)

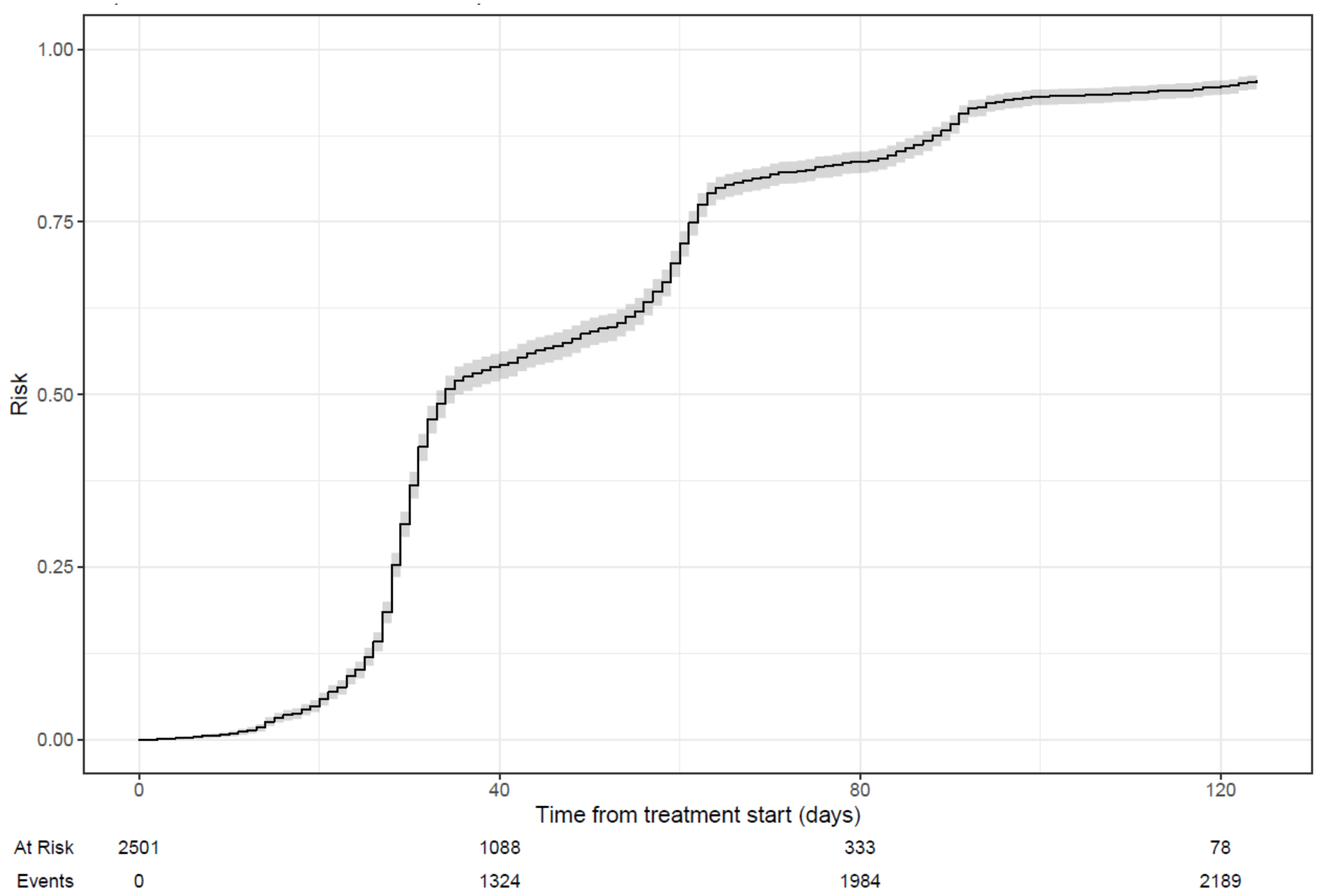

Figure 4. Cumulative probability of remaining in the study after successful treatment outcomes

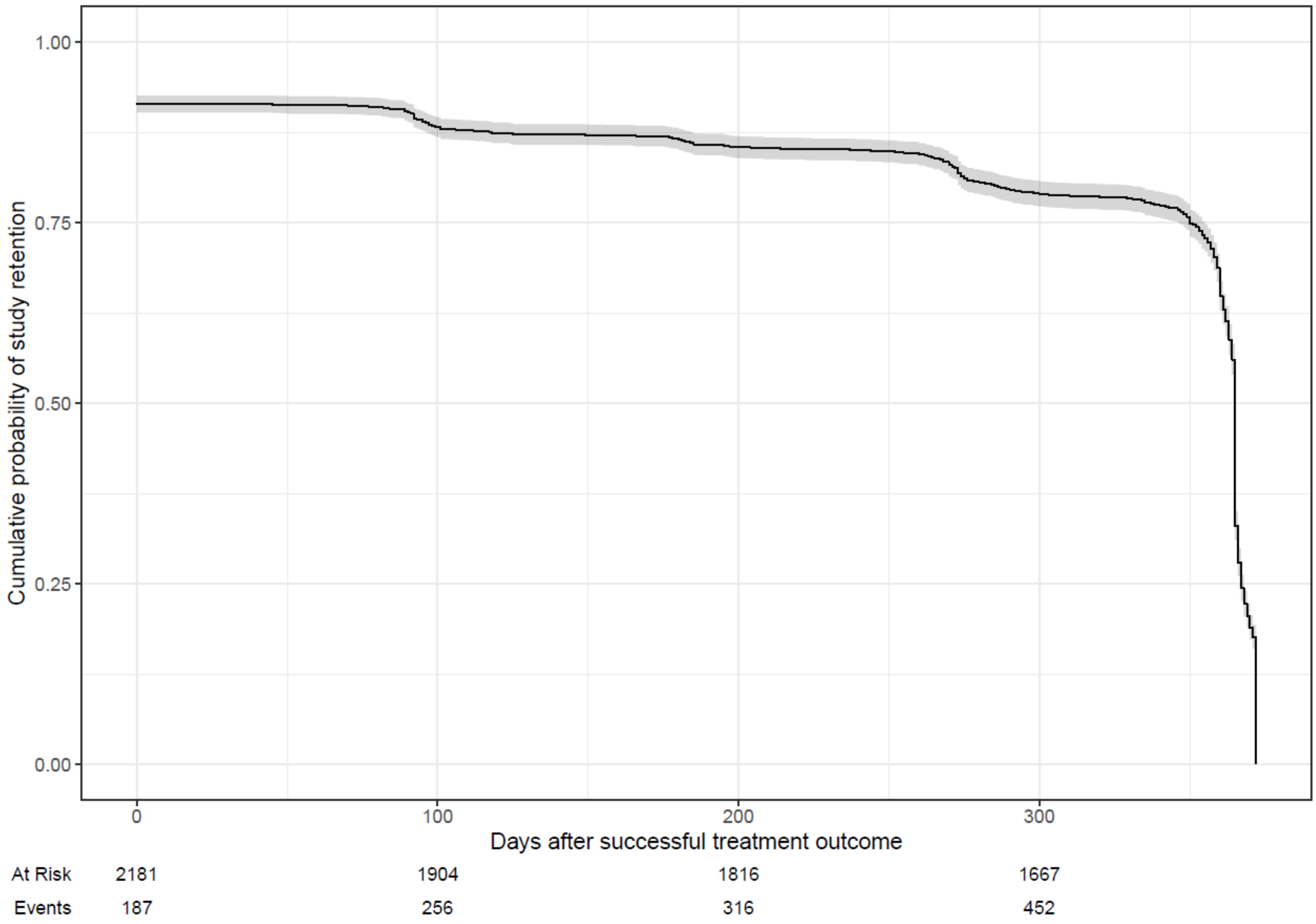

Figure 5. Kaplan-Meier estimates for time to unsuccessful study outcome.

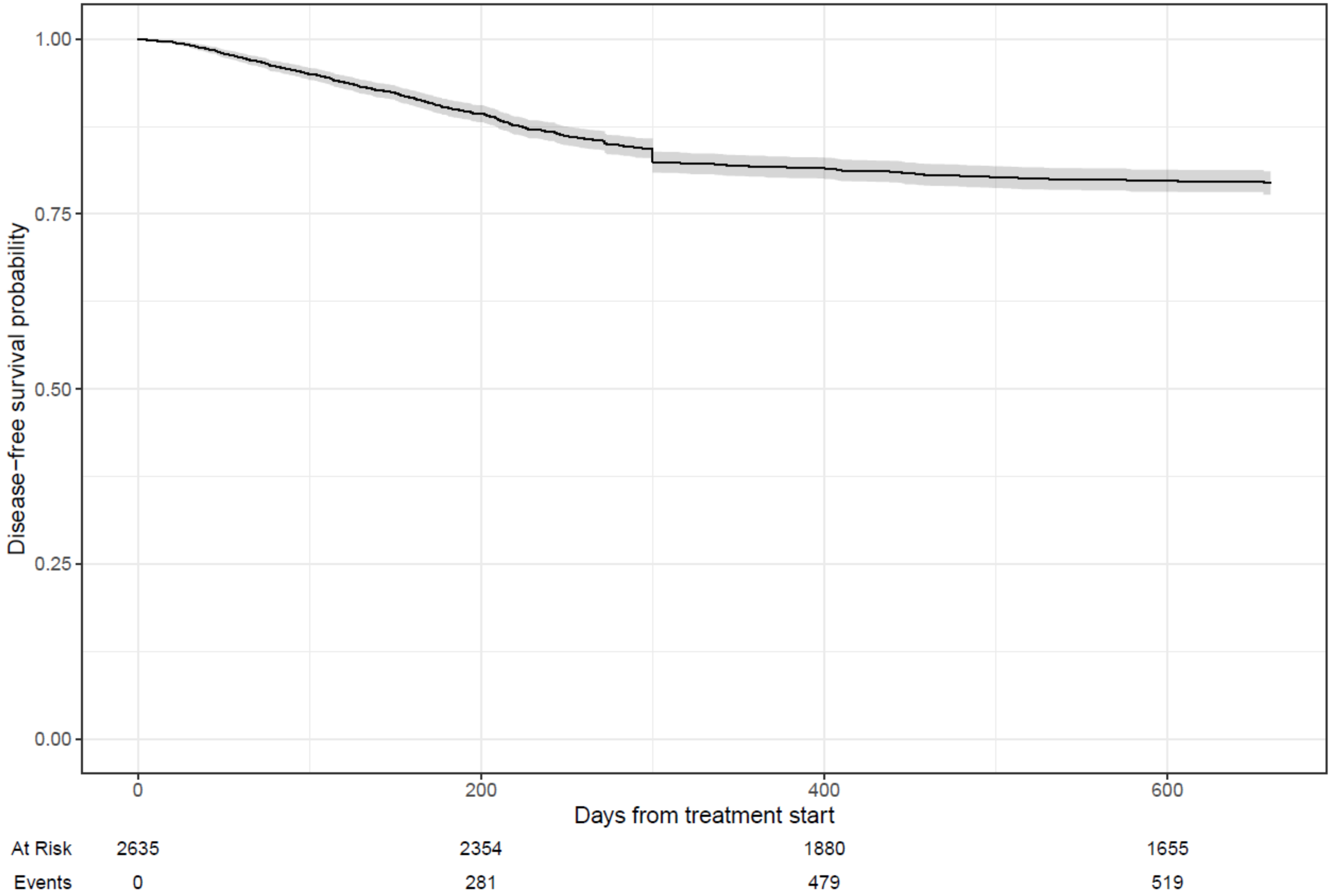

Table 1. List of ethics review bodies that approved mSTR operational research in 2020-2021 and enrollment start dates.

Country	Name of IRB	Date of approval	Name of the Chairperson	Principal Investigator	Enrollment start dates
**Armenia**	Ethics Review Committee Healthcare Research & Development Initiative of Armenia	5 May 2020	Lilit Khachatryan	Dr Naira Khachatryan	28 August 2020
**Azerbaijan**	Ethics Review Committee of the Azerbaijan Medical Institute	18 May 2020	Rauf Baylarov	Dr Irada Akhundova	26 October 2020
**Belarus**	Ethics Review Committee of the State Institution 'Republican Scientific-Practical Center of Pulmonology and Phthisiology'	10 December 2019	T.M. Kritskaya	Prof Alena Skrahina	1 November 2020
**Georgia**	Ethics Committee of the National Center for Tuberculosis and Lung Diseases of Georgia	18 September 2020	Paata Aladashvili	Prof Nana Kiria	4 February 2021
**Kazakhstan**	Ethics Review Committee of the National Scientific Center for Phthisiopulmonology of the Republic of Kazakhstan	11 September 2020	G.A. Smailova	Dr Malik Adenov	12 October 2020
**Kyrgyzstan**	Ethics Committee of the Scientific and Production Center for Preventive Medicine of the Ministry of Health of the Republic of Kyrgyzstan	21 December 2020	D.A. Baiyzbekova	Dr Marat Abdiev	17 February 2021
**Latvia**	Riga Stradins University Ethics Review Committee	10 September 2020	Olaf Bruvers	Dr Liga Kuksa	10 March 2021
**Lithuania**	Kaunas Regional Biomedical Research Ethics Committee	8 January 2021	Gintautas Gumbrevičius	Dr Saulius Diktanas	25 May 2021

**Republic of Moldova**	Committee of Biomedical Ethic of Public Medical Sanitary Institute of Phthisiopneumology "Chiril Draganiuc"	15 May 2020	Valeriu Djugostran	Dr Valentina Vilc	15 September 2020
**Tajikistan**	Biomedical Ethics Committee of the Ministry of Health and Social Protection of Republic of Tajikistan, Academy of Medical Sciences	16 September 2020	M.S. Rustamova	Dr Sadullo Saidaliev	4 December 2020
**Turkmenistan**	Local Ethics Review Committee at the Centers for Infectious Diseases Directorate, Ministry of Health and Medical Industry of Turkmenistan	3 November 2020	B. Jumayev	Dr Mahri Durdyyeva	1 February 2021
**Ukraine**	Committee on Medical Ethics State Institution "National institute of phthisiology and pulmonology named after F. G. Yanovsky, National Academy of Medical Science of Ukraine	14 August 2020	V.I. Korzhov	Dr Iana Terleieva	1 November 2020
**Uzbekistan**	Ethical Committee of the Ministry of Health of Republic of Uzbekistan	12 June 2020	U.K. Kayumov	Dr Nargiza Parpiyeva	19 November 2020

IRB = Institutional Review Board

Table 2. Dosing of medicines used in MDR/RR-TB regimens by weight band in patients older than 14 yearsa.

Group	Medicine	Weight-based daily dose	Formulation	Weight bands for patients older than 14 years[a]					Usual upper daily dose[b]
				30–35 kg	36–45 kg	46–55 kg	56–70 kg	>70 kg	
A	Levofloxacin	-[c]	250 mg tab	3	3	4	4	4	1.5 g
			500 mg tab	1.5	1.5	2	2	2	
			750 mg tab	1	1	1.5	1.5	1.5	
	Bedaquiline	-[c]	100 mg tab	4 tabs od for first 2 weeks; then 2 tabs od M/W/F for 22 weeks					400 mg
	Linezolid	-[c]	600 mg tab	(<15 y)	(<15 y)	1	1	1	1.2 g
B	Clofazimine	-[c]	50 mg cap	2	2	2	2	2	100 mg
			100 mg cap	1	1	1	1	1	100 mg
	Cycloserine or terizidone	10–15 mg/kg	250 mg cap	2	2	3	3	3	1 g
C	Delamanid	-[c]	50 mg tab	2 bd	2 bd	2 bd	2 bd	2 bd	200 mg

bd = two times a day; cap = capsule; g = gram; kg = kilogram; mg = milligram; M/W/F = Monday, Wednesday, Friday; tab = tablet; y=year

Footnotes

[a] Dosages were established by the Guideline Development Group for the *WHO treatment guidelines for rifampicin- and multidrug-resistant tuberculosis, 2018 update* and the WHO Global task force on the pharmacokinetics and pharmacodynamics (PK/PD) of TB medicines and other experts. They are based on the most recent reviews and best practices in the treatment of MDR/RR-TB. For certain agents the dosages were informed by pharmacokinetic modelling results based on the principle of allometric scaling (Anderson BJ, Holford NH. Mechanism-based concepts of size and maturity in pharmacokinetics. Annu Rev Pharmacol Toxicol 2008;48:303–32). Due to the pharmacokinetic properties of certain medicines the doses proposed may exceed the mg/kg/day ranges shown here in order to achieve blood concentrations similar to target levels in an average adult patient. In patients <30 kg follow the schedule for <15 year olds unless otherwise indicated. If multiple dose options are given for one weight band select the lower or higher option depending on whether the patient is at the lower or higher limit of the body weight range. Dosing more closely to the target mg/kg/day should be aimed for, and is more feasible with oral or parenteral fluids and when solid forms of different dosages are available. Fractioning of tablets into halves or less should be avoided, if possible. Therapeutic drug monitoring is advised when the dose is at the upper and lower ends of the range to minimize the adverse therapeutic consequences of over- and under-exposure, respectively (especially for injectable agents, linezolid and fluoroquinolones).

[b] Clinicians may decide to exceed these values in particular cases to improve therapeutic effect.

[c] No weight-based dosing is proposed.

Table 3. Dosing of medicines used in MDR-TB regimens by weight band in patients under 15 years[a]

Group	Medicine	Weight-based daily dose[b]	Formulation	Weight bands among patients not yet 15 years old[a]							Usual upper daily dose[b]	Comments
				5–6 kg	7–9 kg	10–15 kg	16–23 kg	24–30 kg	31–34 kg	>34 kg		
A	Levofloxacin	15–20 mg/kg	100 mg dt	1	1.5	2 or 3	3 or 4	(>14 y)	(>14 y)	(>14 y)	1.5 g	
			250 mg tab	0.5	0.5	1 or 1.5	1.5 or 2	2	3	(>14 y)	1.5 g	
	Bedaquiline	-	100 mg tab	-	-	-	2 tabs od for two weeks; then 1 tab od M/W/F for 22 weeks		4 tabs od for 2 weeks; then 2 tabs od M/W/F for 22 weeks		-	Only in patients >5 years old (lower dose from 15–29 kg; higher dose from >29 kg)
	Linezolid	15 mg/kg od in <16 kg<br>10–12 mg/kg od in >15 kg	20 mg /ml susp	4 ml	6 ml	8 ml	11 ml	14 ml	15 ml	20 ml[d]	600 mg	
			600 mg tab[c]	0.25	0.25	0.25	0.5	0.5	0.5	0.75[d]		
B	Clofazimine	2–5 mg/kg	50 mg cap	1 alt days M/W/F	1 alt days M/W/F	1 alt days	1	2	2	(>14 y)	100 mg	Give on alternate days if dose in mg/kg/day is too high
			100 mg cap			1 alt days	1 alt days	1	(>14 y)	(>14 y)	100 mg	
	Cycloserine or terizidone	15–20 mg/kg	125 mg mini capsule (cycloserine)	1	1	2	3	4	(>14 y)	(>14 y)	1 g	
			250 mg cap[c]	4–5 ml[c]	5–6 ml[c]	7–10 ml[c]	2	2	2	(>14 y)	1 g	
C	Delamanid	-	50 mg tab	-	-[e]	-[e]	-[e]	1 bd	1 bd	2 bd	200 mg	Only in patients >2 years old (25 mg bd in

						3–5 years; 50 mg bd in 6–11 years; 100 mg bd in 12–17 years)

alt=alternate; bd = two times a day; cap = capsule; dt = dispersible tablet; g = gram; kg = kilogram; ml = millilitre; mg = milligram; M/W/F = Monday, Wednesday, Friday; susp = suspension; tab = tablet; y=year

Footnotes

a Dosages were established by the Guideline Development Group for the *WHO treatment guidelines for rifampicin- and multidrug-resistant tuberculosis, 2018 update* and the WHO Global task force on the pharmacokinetics and pharmacodynamics (PK/PD) of TB medicines and other experts. They are based on the most recent reviews and best practices in the treatment of MDR/RR-TB. For certain agents the dosages were informed by pharmacokinetic modelling results based on the principle of allometric scaling (Anderson BJ, Holford NH. Mechanism-based concepts of size and maturity in pharmacokinetics. Annu Rev Pharmacol Toxicol 2008;48:303–32). Due to the pharmacokinetic properties of certain medicines the doses proposed may exceed the mg/kg/day ranges shown here in order to achieve blood concentrations similar to target levels in an average adult patient. In patients >30 kg follow the schedule for >14 year olds unless otherwise indicated. If multiple dose options are given for one weight band select the lower or higher option depending on whether the patient is at the lower or higher limit of the body weight range. Dosing more closely to the target mg/kg/day should be aimed for, and is more feasible with oral or parenteral fluids and when solid forms of different dosage are available. Fractioning of tablets into halves or less should be avoided if possible. Therapeutic drug monitoring is advised when the dose is at the upper and lower ends of the range to minimize the adverse therapeutic consequences of over- and under-exposure respectively (especially for injectable agents, linezolid and fluoroquinolones).

b Clinicians may decide to exceed these values in particular cases to improve therapeutic effect.

c Dissolving in 10 ml of water may facilitate administration in patients in lower weight-bands and avoids fractioning solid formulations, although bioavailability is uncertain (use of dispersible tablets is preferred if available).

d In individuals >44 kg a dose of 600 mg od is proposed.

Table 4. Clinical and bacteriological monitoring schedule for MDR/RR-TB patients on mSTR.

	Baseline Visit	Week 2	Month 1	Month 2	Month 3	Month 4	Month 5	Month 6	Until end of treatment	End of treatment	3 months post-end-of-treatment	6 months post-end-of-treatment	9 months post-end-of-treatment	12 months post-end-of-treatment
Clinical evaluation														
Vital signs	×		×	×	×	×	×	×	Monthly	×	×	×	×	×
Brief peripheral neuropathy screen	×		×	×	×	×	×	×	Monthly	×				×
Visual acuity and colorblindness screen	×		×	×	×	×	×	×	Monthly	×				×
Post-end-of- treatment consultation										×	×	×	×	×
Assessment and follow-up of adverse events	×	×	×	×	×	×	×	×	At each scheduled /unscheduled visit	×	×	×	×	×
Weight	×	×	×	×	×	×	×	×	Monthly	×	×	×	×	×
Bacteriological testing														
Smear	×		×	×	×	×	×	×	Monthly	×		×		×
Culture	×		×	×	×	×	×	×	Monthly	×		×		×
Freeze baseline culture	×													
Xpert MTB/RIF	×													
LPA (Hain GenoType MTBDRsl)	×			If smear- or culture-positive check for amplification of resistance										
Culture-based first-line DST	×			If smear- or culture-positive check for amplification of resistance										
Culture-based second-line DST	×			If smear- or culture-positive check for amplification of resistance										

	Baseline Visit	Week 2	Month 1	Month 2	Month 3	Month 4	Month 5	Month 6	Until end of treatment	End of treatment	3 months post-end-of-treatment	6 months post-end-of-treatment	9 months post-end-of-treatment	12 months post-end-of-treatment
Laboratory testing														
ECG	×	×	×	×	×	×	×	×	Monthly	×	×	×		
Full blood count (hemoglobin, red blood cells. white blood cells and platelets) if on Lzd	×		×	×	×	×	×	×	Monthly (if on Lzd)	×				
Liver function tests (AST, ALT)	×		×	×	×	×	×	×	Monthly	×				
Serum creatinine	×		×	×	×	×	×	×	Monthly	×				
Serum potassium	×		×	×	×	×	×	×	Monthly	×				
Hepatitis Bs Antigen	×													
Hepatitis C Antibody	×													
HbA1c	×		Repeated every 3 months if elevated											
COVID-19 PCR	×		At baseline and then only if clinically indicated											
Pregnancy test (females)	×													
HIV testing	×													
CD4 (repeated every 6 months if HIV+)	×							×						
HIV Viral load (repeated every 6 months if HIV+)	×							×						
Chest X-Ray	×							×		×				

LPA= line probe assay; DST=drug sensitivity test; ECG=electrocardiogram; Lzd=linezolid; ALT=alanine aminotransferase. AST=aspartate aminotransferase; HbA1c=HbA1c; PCR=Polymerase chain reaction; HIV=human immunodeficiency virus.

Table 5. Key definitions used in the operational research on mSTR.

EVENT	DEFINITION
**Cured**	A patient with bacteriologically confirmed RR-TB who has completed treatment as recommended by this protocol without evidence of failure AND at least three or more consecutive cultures taken at least 30 days apart are negative at the end of treatment
**Treatment completed**	Treatment completed as recommended by this protocol without evidence of failure BUT no record that three or more consecutive cultures taken at least 30 days apart are negative at the end of treatment
**Treatment failed**	Treatment terminated or need for permanent regimen change of at least two anti-TB drugs because of:<br>• lack of sputum culture conversion after 4 months of treatment, or<br>• bacteriological reversion of sputum culture after 5 months of treatment in a patient with previous culture conversion to negative, or<br>• evidence of additional acquired resistance to fluoroquinolones, Bdq, Dlm, Lzd, Cfz, or<br>• adverse drug reactions (leading to the change of at least two anti-TB drugs in the regimen)<br>• failed to receive 246-301 doses of treatment regimen within up to 43 weeks regardless of the reason (unless they satisfied the definition of lost to follow-up)<br>• received more than 301 doses of treatment regimen regardless of the reason
**Died**	A patient who dies for any reason during the course of treatment and 12 months follow-up period
**Lost to follow-up**	A patient whose treatment was interrupted for 2 consecutive months or more
**Not evaluated for end of study outcome**	A patient whose sustained treatment success was not evaluated at 12 months of follow-up
**Withdrawn**	A patient is taken off the mSTR for any reason other than treatment failure (for example, baseline second-line drug resistance, withdrawn patient informed consent, or other reasons) and referred to the PMDT programme for routine care
**Treatment success**	The sum of *cured* and *treatment completed*
**Recurrence**	Cure or treatment completion with two consecutive positive cultures during post-treatment follow-up, or one positive culture with clinical signs and symptoms or radiographic deterioration, but without genotyping information on baseline and recurrent strain
**Conversion (to negative)**	Culture is considered to have converted to negative when two consecutive cultures taken at least 30 days apart are found to be negative. In such case, the specimen collection date of the first negative culture is used as the date of conversion.

TB=tuberculosis; RR-TB=rifampicin-resistant TB, Bdq=bedaquiline; Cfz=clofazimine; Dlm=delamanid; Lzd=linezolid; mSTR=modified short treatment regimens; PMDT= programmatic management of drug-resistant TB.

Table 6. Participants' baseline demographic and clinical characteristics by each study regimen.

Characteristic	Lfx + Bdq + Lzd + Cfz + Cs, N = 2532[1]	Lfx + Bdq + Lzd + Cfz + Dlm, N = 75[1]	Lfx + Dlm + Lzd + Cfz, N = 29[1]
**Age (yrs)**	43 (34, 53)	41 (34, 53)	6 (4, 10)
**Age group (yrs)**			
<15	19 (0.8%)	1 (1.3%)	27 (93.1%)
15-24	188 (7.4%)	4 (5.3%)	2 (6.9%)
35-44	700 (27.6%)	25 (33.3%)	0 (0.0%)
45-54	578 (22.8%)	14 (18.7%)	0 (0.0%)
55-64	419 (16.5%)	10 (13.3%)	0 (0.0%)
>64	147 (5.8%)	4 (5.3%)	0 (0.0%)
Sex			
Male	1899 (75.0%)	49 (65.3%)	18 (62.1%)
Female	633 (25.0%)	26 (34.7%)	11 (37.9%)
Body Mass Index (kg/m^2)			
<18.5	538 (21.2%)	16 (21.3%)	26 (89.7%)
≥18.5	1994 (78.8%)	59 (78.7%)	3 (10.3%)
Country			
Armenia	25 (1.0%)	3 (4.0%)	0 (0.0%)
Azerbaijan	101 (4.0%)	0 (0.0%)	0 (0.0%)
Belarus	535 (21.1%)	5 (6.7%)	0 (0.0%)
Georgia	96 (3.8%)	3 (4.0%)	1 (3.4%)
Kazakhstan	145 (5.7%)	3 (4.0%)	0 (0.0%)
Kyrgyzstan	82 (3.2%)	0 (0.0%)	11 (37.9%)
Latvia	16 (0.6%)	1 (1.3%)	0 (0.0%)
Lithuania	8 (0.3%)	0 (0.0%)	0 (0.0%)
Republic of Moldova	103 (4.1%)	1 (1.3%)	0 (0.0%)
Tajikistan	97 (3.8%)	0 (0.0%)	5 (17.2%)
Turkmenistan	107 (4.2%)	1 (1.3%)	0 (0.0%)
Ukraine	1029 (40.6%)	58 (77.3%)	11 (37.9%)
Uzbekistan	188 (7.4%)	0 (0.0%)	1 (3.4%)
Inclusion cohort			

**Characteristic**	**Lfx + Bdq + Lzd + Cfz + Cs,** N = 2532[1]	**Lfx + Bdq + Lzd + Cfz + Dlm,** N = 75[1]	**Lfx + Dlm + Lzd + Cfz,** N = 29[1]
Historical	324 (12.8%)	8 (10.6%)	1 (3.4%)
Prospective	2208 (87.2%)	67 (88.4%)	28 (96.6%)
History of injecting drug use			
No	2450 (97.3%)	71 (95.9%)	29 (100.0%)
Yes	68 (2.7%)	3 (4.1%)	0 (0.0%)
Missing	14	1	0
Homeless			
No	2,454 (97.3%)	72 (96.0%)	29 (100.0%)
Yes	67 (2.7%)	3 (4.0%)	0 (0.0%)
Missing	11	0	0
Employment status			
Employed	568 (22.4%)	25 (33.3%)	0 (0.0%)
Unemployed	1562 (61.7%)	41 (54.7%)	0 (0.0%)
Other	402 (15.9%)	9 (12.0%)	29 (100.0%)
History of incarceration			
No	2273 (89.9%)	71 (94.7%)	29 (100.0%)
Yes	254 (10.1%)	4 (5.3%)	0 (0.0%)
Missing	5	0	0
Smoking history			
No	1156 (45.8%)	39 (52.0%)	28 (96.6%)
Yes	1368 (54.2%)	36 (48.0%)	1 (3.4%)
Missing	8	0	0
Excess alcohol use			
No	2103 (83.8%)	64 (87.7%)	29 (100.0%)
Yes	406 (16.2%)	9 (12.3%)	0 (0.0%)
Missing	23	2	0
HIV positive status			
No	2270 (89.8%)	65 (86.7%)	28 (96.6%)
Yes	259 (10.2%)	10 (13.3%)	1 (3.4%)
Missing	3	0	0
HCV Ab status			
Seronegative	2129 (88.3%)	64 (85.3%)	18 (100.0%)

**Characteristic**	**Lfx + Bdq + Lzd + Cfz + Cs**, N = 2532[1]	**Lfx + Bdq + Lzd + Cfz + Dlm**, N = 75[1]	**Lfx + Dlm + Lzd + Cfz**, N = 29[1]
Seropositive	281 (11.7%)	11 (14.7%)	0 (0.0%)
Missing	122	0	11
Diabetes			
No	2315 (91.5%)	71 (94.7%)	29 (100.0%)
Yes	216 (8.5%)	4 (5.3%)	0 (0.0%)
Missing	1	0	0
Baseline SARS-CoV2 status			
No	1946 (96.0%)	66 (97.1%)	24 (100%)
Yes	81 (4.0%)	2 (2.9%)	0 (0.0%)
Missing	505	7	5
Previous TB episode			
No	1903 (75.3%)	53 (70.7%)	27 (93.1%)
Yes	624 (24.7%)	22 (29.3%)	2 (6.9%)
Missing	5	0	0
X-ray cavities			
No cavity	1048 (41.4%)	40 (53.3%)	25 (92.6%)
Unilateral	1048 (41.4%)	23 (30.7%)	2 (7.4%)
Bilateral	433 (17.1%)	12 (16.0%)	0 (0.0%)
Missing	3	0	2
Baseline smear microscopy status			
Negative	1013 (41.1%)	32 (44.4%)	21 (91.3%)
Positive	1454 (58.9%)	40 (55.6%)	2 (8.7%)
Missing	65	3	6
Baseline elevated AST/ALT			
None	1972 (78.0%)	68 (90.6%)	25 (86.2%)
Grade 1	506 (20.0%)	6 (8.0%)	4 (13.8%)
Grade 2	44 (1.7%)	1 (1.3%)	0 (0.0%)
Grade 3	9 (0.4%)	0 (0.0%)	0 (0.0%)
Grade 4	1 (<0.1%)	0 (0.0%)	0 (0.0%)
Baseline peripheral neuropathy			
None	2389 (94.4%)	68 (90.7%)	29 (100.0%)

Characteristic	**Lfx + Bdq + Lzd + Cfz + Cs**, N = 2532[1]	**Lfx + Bdq + Lzd + Cfz + Dlm**, N = 75[1]	**Lfx + Dlm + Lzd + Cfz**, N = 29[1]
Grade 1	114 (4.5%)	6 (8.0%)	0 (0.0%)
Grade 2	24 (0.9%)	1 (1.3%)	0 (0.0%)
Grade 3	5 (0.2%)	0 (0.0%)	0 (0.0%)
Grade 4	0 (0.0%)	0 (0.0%)	0 (0.0%)
Baseline anaemia			
None	2056 (81.2%)	69 (92.0%)	27 (93.1%)
Grade 1	295 (11.7%)	3 (4.0%)	2 (6.9%)
Grade 2	156 (6.2%)	3 (4.0%)	0 (0.0%)
Grade 3	23 (0.9%)	0 (0.0%)	0 (0.0%)
Grade 4	2 (<0.1%)	0 (0.0%)	0 (0.0%)
Baseline renal dysfunction			
None	2403 (94.9%)	71 (94.7%)	29 (100.0%)
Grade 1	126 (5.0%)	3 (4.0%)	0 (0.0%)
Grade 2	2 (<0.1%)	1 (1.3%)	0 (0.0%)
Grade 3	1 (<0.1%)	0 (0.0%)	0 (0.0%)
Grade 4	0 (0.0%)	0 (0.0%)	0 (0.0%)
Baseline visual loss			
None	1935 (76.4%)	59 (78.7%)	28 (96.6%)
Grade 1	172 (6.8%)	8 (10.7%)	1 (3.4%)
Grade 2	232 (9.2%)	5 (6.7%)	0 (0.0%)
Grade 3	130 (5.1%)	3 (4.0%)	0 (0.0%)
Grade 4	63 (2.5%)	0 (0.0%)	0 (0.0%)

Data are median (IQR), n (%); AFB=acid fast bacilli; ALT=alanine aminotransferase; AST=aspartate aminotransferase; Bdq=bedaquiline; Cfz=clofazimine; Cs=cycloserine; Dlm=delamanid; HCV Ab=Hepatitis C virus antibody; Lfx=levofloxacin; Lzd=linezolid; HIV=human immunodeficiency virus.

Table 7. End of treatment outcomes by study regimen.

	**Total,** N=2636	**Lfx + Bdq + Lzd + Cfz + Cs,** N = 2532	**Lfx + Bdq + Lzd + Cfz + Dlm,** N = 75	**Lfx + Dlm + Lzd + Cfz,** N = 29
Cured	1649 (62.6%)	1606 (63.4%)	41 (54.7%)	2 (6.9%)
Completed	532 (20.2%)	487 (19.2%)	20 (26.6%)	25 (86.2%)
Failed	191 (7.2%)	184 (7.3%)	5 (6.7%)	2 (6.9%)
Died	113 (4.3%)	110 (4.3%)	3 (4.0%)	0 (0%)
Lost to follow-up	150 (5.7%)	144 (5.7%)	6 (8.0%)	0 (0%)
Missing	1	1	0	0
Treatment success	2181 (82.7%)	2093 (82.7%)	61 (81.3%)	27 (93.1%)

Bdq=bedaquiline; Cfz=clofazimine; Cs=cycloserine; Dlm=delamanid; Lfx=levofloxacin; Lzd=linezolid.

Table 8. Reasons for treatment failure with mSTR.

Reasons for treatment failure	N = 191[1]
Adverse drug reaction resulting in treatment change	32 (24.0%)
Treatment extended by clinician	31 (23.3%)
Insufficient treatment received in accepted time	24 (18.0%)
Failure to culture convert	16 (12.0%)
Acquired drug resistance	11 (8.3%)
Clinical deterioration	11 (8.3%)
Culture reverted	8 (6.0%)
Unknown	58

[1]n (%)

Table 9. End of treatment outcomes for all participants by baseline HIV status.

End of treatment outcome	HIV status	
	**Negative**, N = 2363[1]	**Positive**, N = 270[1]
Cured	1548 (65.5%)	100 (37.2%)
Completed	444 (18.8%)	87 (32.3%)
Failed	166 (7.0%)	24 (8.9%)
Died	84 (3.6%)	29 (10.8%)
Lost to follow-up	121 (5.1%)	29 (10.8%)
Missing	0	1
Treatment success	1992 (84.3%)	187 (69.3%)

[1]n (%); HIV= human immunodeficiency virus.

Table 10. Crude associations between baseline HIV treatment and CD4 count and time to unsuccessful study outcome and time to death in the treatment cohort.

	Unsuccessful study outcome					Death				
**Characteristic**	**N**	**Event N**	**HR**	**95% CI**	**p-value**	**N**	**Event N**	**HR**	**95% CI**	**p-value**
Baseline ART status	268					268				
No		16	—	—			9	—	—	
Yes		78	0.63	0.37, 1.09	0.10		30	0.43	0.20, 0.90	0.026
Receiving cotrimoxazole	262					262				
Yes		88	—	—			36	—	—	
No		5	2.69	1.09, 6.63	0.032		3	3.80	1.17, 12.4	0.027
Baseline CD4 group	245					245				
0-100		32	—	—			18	—	—	
101-250		31	1.25	0.76, 2.06	0.37		12	0.85	0.41, 1.76	0.66
251-500		15	0.67	0.36, 1.24	0.2		3	0.23	0.07, 0.79	0.019
500+		6	0.40	0.17, 0.96	0.040		2	0.23	0.05, 0.99	0.048

HR = Hazard Ratio; CI = Confidence Interval; HIV=human immunodeficiency virus; ART=antiretroviral therapy.
